# Overexpression of Douglas-Fir *LEAFY COTYLEDON1* (*PmLEC1*) in *Arabidopsis* Induces Embryonic Programs and Embryo-like Structures in the *lec1-1* Mutant but Not in Wild Type Plants

**DOI:** 10.3390/plants10081526

**Published:** 2021-07-26

**Authors:** Mariana A. Vetrici, Dmytro P. Yevtushenko, Santosh Misra

**Affiliations:** 1 Department of Biological Sciences, University of Lethbridge, 4401 University Drive, Lethbridge, AB T1K 3M4, Canada; dmytro.yevtushenko@uleth.ca; 2 Centre for Forest Biology, Department of Biochemistry and Microbiology, University of Victoria, Victoria, BC V8W 2Y2, Canada; misras@telus.net

**Keywords:** Douglas-fir, somatic embryogenesis, *LEAFY COTYLEDON1*, transformation, *lec1-1* null mutant, embryo-specific gene expression, anti-PmLEC1 antibodies

## Abstract

Somatic embryogenesis (SE) is the most promising method for the quick propagation of desirable plant genotypes. However, application of SE to conifers remains challenging due to our limited knowledge about the genes involved in embryogenesis and the processes that lead to somatic embryo formation. Douglas-fir, an economically important lumber species, possesses a homolog of the angiosperm embryo-regulatory *LEC1* gene. In the present study, we analyzed the potential of Douglas-fir *PmLEC1* to induce embryonic programs in the vegetative cells of a heterologous host, *Arabidopsis thaliana*. *PmLEC1* complemented the *Arabidopsis lec1-1* null mutant and led to a variety of phenotypes ranging from normal morphology to developmental arrest at various stages in the T1 generation. *PmLEC1* did not affect the morphology of wild type *Arabidopsis* T1 plants. More profound results occurred in T2 generations. *PmLEC1* expression induced formation of recurrent somatic embryo-like structures in vegetative tissues of the rescued *lec1-1* mutant but loss of apical dominance (bushy phenotype) in wild type plants. The activation of embryonic programs in the *lec1-1^PmLEC1^* T2 plants was confirmed by the presence of the embryo-specific transcripts, *OLEOSIN* and *CRUCIFERIN*. In contrast, no embryo-like structures, and no *OLEOSIN* or *CRUCIFERIN* were observed in *PmLEC1*-expressing bushy wild type T2 plants.

## 1. Introduction

Douglas-fir (*Pseudotsuga menziesii* [Mirb.] Franco) is a superior softwood species that is native to Western North America but cultivated throughout Europe, Australia, New Zealand and South America due to its desirable wood qualities and ability to withstand biotic and abiotic stresses. Somatic embryogenesis (SE) is the most promising method for meeting reforestation and afforestation demands of Douglas-fir because seed production in this species is limited and uncertain, germination efficiencies are low and reproductive cycles are long (17 months). The recalcitrance of coniferous species to induction of SE and the paucity of knowledge regarding the molecular events responsible for gymnosperm embryogenesis [1] are driving forces toward the identification and functional characterization of embryo-regulatory genes. In angiosperms, hundreds of genes that function during embryogenesis have been identified and characterized, with *LEC1* standing out as the most promising for SE induction. The function of *LEC1* in embryogenesis was initially established via the rescue of *Arabidopsis thaliana lec1-1* null mutants with the *Arabidopsis LEAFY COTYLEDON1* (*AtLEC1*) gene [2]. The *lec1-1* null mutant is embryo-lethal; however, *lec1-1* embryos can be rescued in vitro prior to desiccation and germinated to produce fertile, flowering plants with normal phenotype [2,3,4]. Integration of the *AtLEC1* gene into the genome of the *lec1-1* null mutant complements the mutation and results in the development of desiccation-tolerant seeds [2]. Furthermore, ectopic expression of *AtLEC1* in the *Arabidopsis lec1-1* null mutant induces embryonic programs and spontaneous formation of somatic embryos from vegetative tissues [2]. *AtLEC1* is necessary for proper zygotic embryo formation and maturation [2,3], and induction of SE in vitro [5]. The absolute requirement for *LEC1* during embryogenesis is demonstrated by the inability of *lec1-1* null mutants to develop somatic embryos in the presence of 2,4-dichlorophenoxyacetic acid (2,4-D), a growth regulator that efficiently induces SE in vitro in wild type *Arabidopsis* [5]. The characterization and functional analysis of *LEC1* genes in conifers will provide critical information for improving SE protocols that facilitate efficient mass production of robust somatic embryos and seedlings for reforestation programs.

We previously reported the isolation and molecular characterization of the *PmLEC1* gene from Douglas-fir [6], a homolog of the embryo-regulatory *LEC1* gene in angiosperms. The aim of the current study was to evaluate the ability of *PmLEC1* to induce embryonic programs and to identify the downstream effects of *PmLEC1* expression in the host plant. To achieve this, we transformed *A. thaliana* Wassilewskija ecotype wild type and *lec1-1* null mutant plants with the *PmLEC1* transgene under the control of a strong constitutive promoter. We also transformed wild type and *lec1-1* null mutant plants with *AtLEC1*. The *lec1-1* null mutant is a homozygous deletion mutant lacking the *LEC1* gene and the *LEC1* promoter [2,4]. This mutation is embryo-lethal because its seeds are desiccation intolerant, thus not viable. However, complementation of the *lec1-1* null mutation and rescue of the mutant is possible when *LEC1* transgenes are introduced into the plant prior to seed desiccation [2,7]. The first transgenic generation (T1) represents transgene integration at fertilization, while the second transgenic generation (T2) represents seeds and plants in which the transgene is present throughout the lifecycle. We found that the ectopic expression of *PmLEC1* complemented the *Arabidopsis lec1-1* null mutant and resulted in the production of T2 seedlings with both normal and embryo-like characteristics, but the expression of embryo-specific genes was limited to the embryo-like seedlings. Spontaneous formation of somatic embryo-like structures on vegetative tissues was also observed. These results indicate that, similarly to *AtLEC1*, *PmLEC1* rescued the *lec1-1* null mutant, and its ectopic expression induced embryonic programs and the formation of embryo-like structures. Overexpression of *PmLEC1* in wild type *Arabidopsis* resulted in bushy T2 plants that expressed *PmLEC1* transcripts in vegetative tissues, but embryonic programs were not induced, and embryo-like structures were not observed. This suggests that the *AtLEC1* promoter, which is absent in the *lec1-1* null mutant but present in wild type *Arabidopsis*, controls the ability of a *LEC1* transgene to induce embryonic programs in vegetative tissues. In addition, the T2 generations exhibited more extreme phenotypes in both the *lec1-1* and wild type genetic backgrounds, indicating that *LEC1* may have important functions prior to fertilization. Western blot analysis demonstrated a strong correlation between phenotype and PmLEC1 accumulation. In summary, *PmLEC1* is likely to be critical in embryogenesis and SE induction; however, overexpression of *LEC1* by itself may not be sufficient to induce SE in mature seeds or from vegetative tissues. PmLEC1 protein levels, rather than *PmLEC1* gene expression, may be more useful in determining embryogenic status and viability of SE cultures.

## 2. Results

To characterize the function of Douglas-fir *LEC1*, we inserted the *PmLEC1* coding sequence into a plant transformation vector, downstream of a strong constitutive promoter *35S-35S-AMV* consisting of the cauliflower mosaic virus (*CaMV*) duplicated enhancer *35S* and the alfalfa mosaic virus untranslated leader sequence *AMV* [8]. The resulting *PmLEC1* gene-expressing construct was transferred into the genomes of wild type and *lec1-1* null mutant *Arabidopsis* plants. In another set of experiments, plants were transformed with the coding sequence of *AtLEC1* under the control of the *35S-35S-AMV* promoter to compare the functions and downstream effects of *Arabidopsis* and Douglas-fir *LEC1* genes in transgenic hosts. We focused on the second generation of transgenic plants (T2) as the appropriate stage for most phenotypical studies.

### 2.1. PmLEC1 Rescues the lec1-1 Null Mutant, Inhibits Vegetative Development and Leads to the Formation of Embryo-like Structures in Abnormal T1 Seedlings

**Figure 1:**
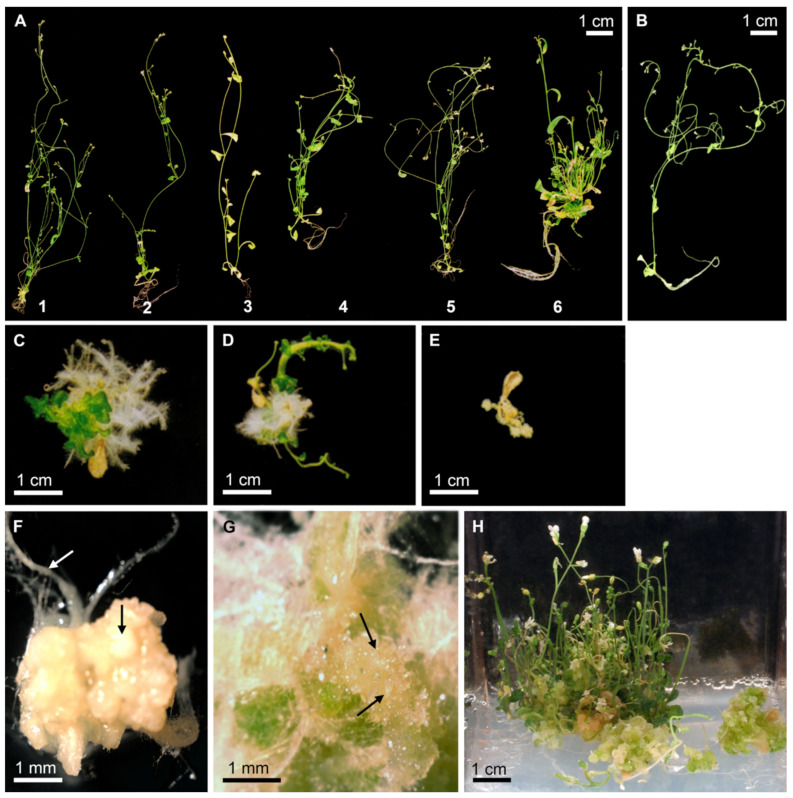
Morphology of *Arabidopsis lec1-1^PmLEC1^* T1 lines containing the Douglas-fir *PmLEC1* transgene. (**A**) Representative T1 lines displaying *Arabidopsis* seedlings with normal morphology (plants 1 to 5) or a bushy phenotype (plant 6). (**B**) Wild type seedling (control, non-transgenic). (**C**) Seedling with multiple cotyledon-like organs. (**D**) Seedling exhibiting fasciation-like symptoms with stunted lateral organs. (**E**) Germinated seedling that stopped further development and died. (**F**) Seedling with normal roots (white arrow) and non-embryogenic callus tissue (black arrow). (**G**) Seedling-derived callus resembling globular stage somatic embryos (arrows). (**H**) Regeneration of morphologically normal plants from an embryogenic callus that proliferated on half-strength MS medium without plant growth regulators for more than one year.

**Figure 2:**
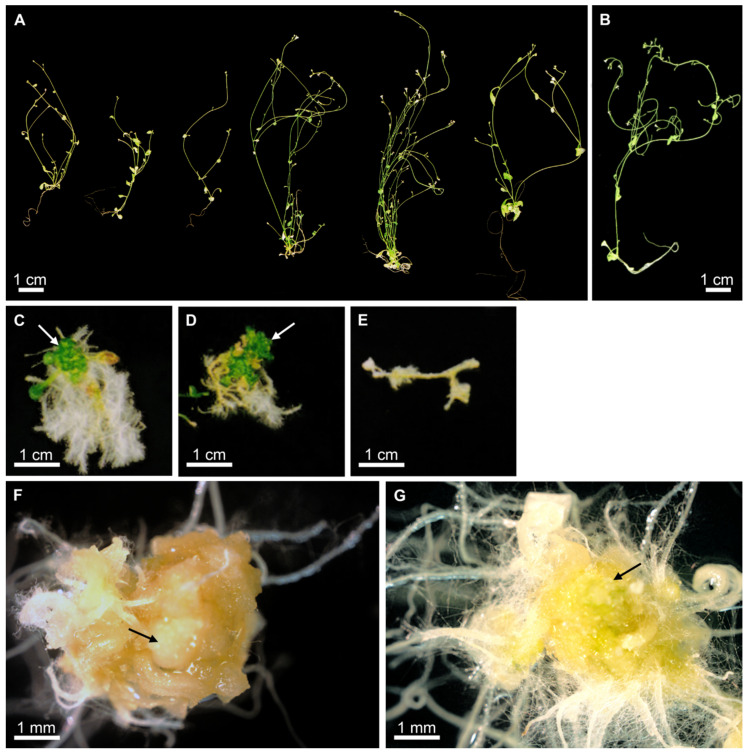
Morphology of *Arabidopsis lec1-1^AtLEC1^* T1 lines containing the *Arabidopsis AtLEC1* transgene. (**A**) Representative T1 lines displaying *Arabidopsis* seedlings with normal morphology. (**B**) Wild type seedling (control, non-transgenic). (**C**,**D**) seedlings with multiple cotyledon-like organs (arrows). (**E**) Germinated seedling that stopped further development and died. (**F**) Seedling-derived non-embryogenic callus with smooth surface (arrow). (**G**) Seedling-derived callus with globular stage somatic embryo-like structures (arrow).

**Figure 3:**
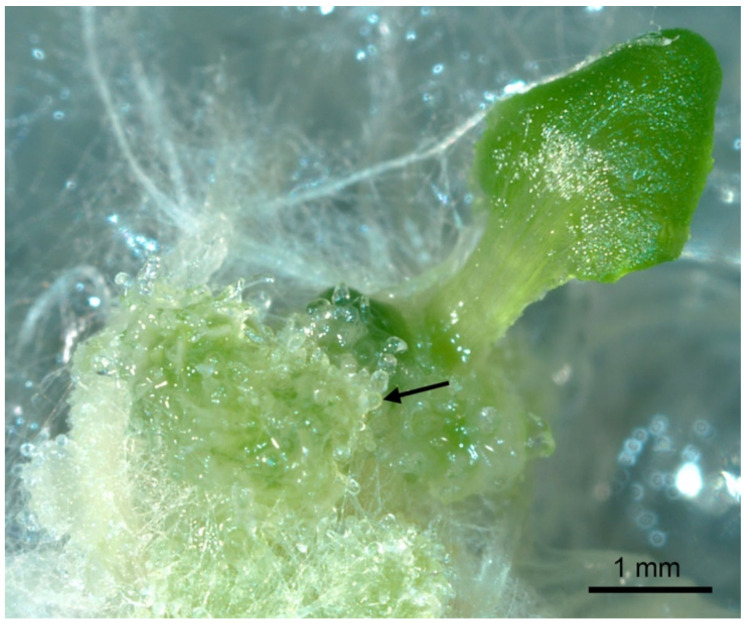
Spontaneous formation of embryo-like structures in T2 generation of *Arabidopsis lec1-1^PmLEC1^* seedling containing the Douglas-fir *PmLEC1* transgene. T2 *lec1-1^PmLEC1^* seeds germinated on kanamycin-containing MS medium and produced roots and cotyledons. Multiple embryo-like structures emerged from one cotyledon, whereas the other cotyledon remained morphologically normal. Several of these structures resembled late globular stage embryos (arrow).

**Figure 4:**
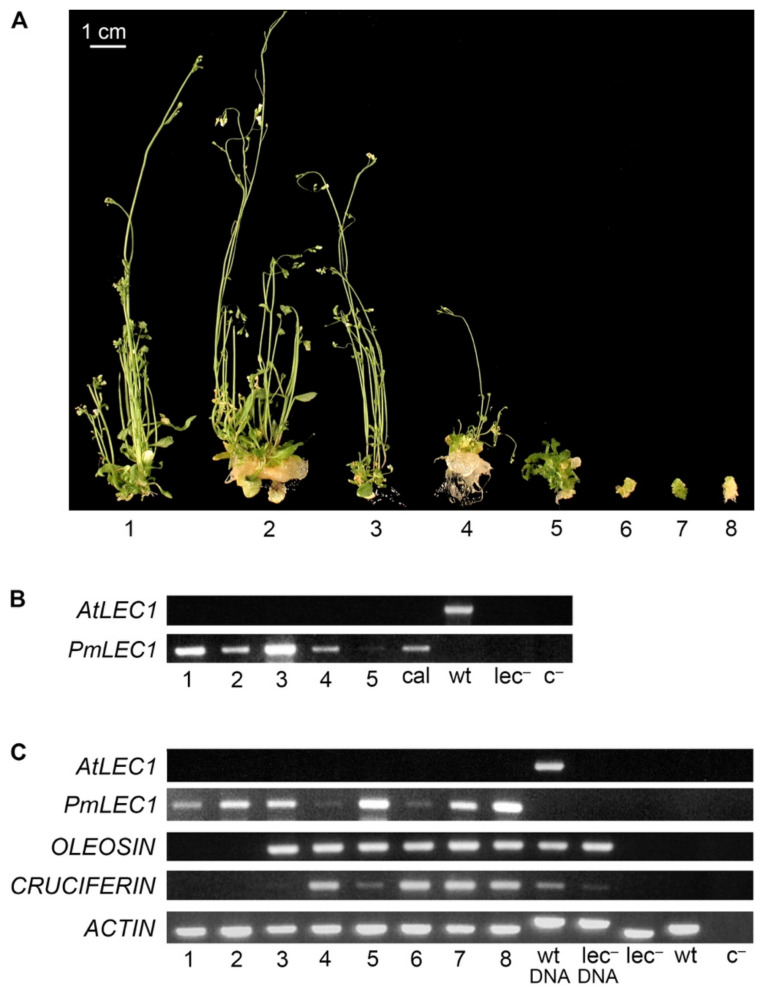
Morphology, genotype analysis and gene expression profiles of individual T2 variants derived from one *lec1-1^PmLEC1^* T1 progenitor line. (**A**) Morphological variations among T2 seedlings derived from a self-pollinated *lec1-1^PmLEC1^* T1 transgenic line. (**B**) PCR analysis of T2 variants using *AtLEC1*- and *PmLEC1*-specific primers (top and bottom panel, respectively) to confirm genotype and the presence of transgenes. Lanes 1 to 5 represent DNA from the plants shown in panel A; cal, DNA from callus tissues morphologically similar to variants 6, 7 and 8 in panel A; wt, genomic DNA from a wild type *Arabidopsis* plant (positive control); *lec*^−^, genomic DNA from a *lec1-1* null mutant plant; c^−^, PCR without DNA template (negative control). (**C**) RT-PCR analysis of gene expression. Each reaction comprised 0.1 μg DNase I-treated total RNA isolated from the indicated plant material. Analyzed genes are shown on the right of the corresponding panels. Lanes 1 to 8 represent transcripts from the plants shown in panel A; wt DNA, genomic DNA from a wild type *Arabidopsis* plant (DNA control); *lec*^−^ DNA, genomic DNA from a *lec1-1* null mutant plant (DNA control); *lec*^−^, RNA from a *lec1-1* null mutant plant; wt, RNA from a wild type *Arabidopsis* plant; c^−^, reverse transcription without RNA, followed by PCR (negative control).

*Arabidopsis lec1-1* null mutant plants transformed with Douglas-fir *PmLEC1* produced viable, desiccation-tolerant T1 seeds that germinated on kanamycin-containing selection MS medium. The floral dip transformation frequency was 1.8%. The *PmLEC1* transgene complemented the null mutation by conferring desiccation tolerance. We confirmed the presence of *PmLEC1* and the absence of *AtLEC1* in the genome of transformed *lec1-1* null mutant seedlings by PCR analysis. Four distinct seedling phenotypes were observed following T1 seed germination (Figure 1): (1) physiologically normal vegetative growth and development (Figure 1A), (2) abnormal, stunted shoot development or fasciation (Figure 1C,D), (3) death (Figure 1E) and (4) callus formation (Figure 1F,G). Normal, wild type plant morphology is shown in Figure 1B.

Approximately 25% (47/200) of germinated T1 seeds produced seedlings with normal morphology (Figure 1A). Of the remaining seeds, ~50% (102/200) produced seedlings that initiated vegetative growth but were developmentally arrested before flowering (Figure 1C–G). These seedlings either had multiple, dark green, cotyledon-like organs (Figure 1C) or thick stems with stunted lateral organs and fleshy leaves (Figure 1D), with the twisted, hooked stem representing extreme fasciation. Fasciation, the abnormal flattening or compression of stems or leaf stalks, results from abnormal development of the meristem as a band-like structure [9]. The remaining ~25% of seedlings (51/200) developed to the stage of two white, fleshy leaves and died (Figure 1E).

A few of the developmentally arrested seedlings formed normal roots, but instead of shoots, a mass of smooth white callus tissue proliferated from the region of the shoot apical meristem (Figure 1F). Other developmentally arrested seedlings formed normal roots and a callus tissue composed of masses of green and colorless globular structures (Figure 1G) resembling somatic embryogenic clusters. These cell masses were maintained on selection MS medium for over one year, with sub-culturing to fresh medium every 4 months. The embryo-like callus proliferated continuously, and after one year, shoots emerged and plants developed (Figure 1H). The decline of embryogenic potential with prolonged sub-culturing has been reported previously [9]; perhaps this phenomenon finally permitted vegetative growth.

The control transformation of *lec1-1* null mutant plants with *Arabidopsis AtLEC1* produced phenotypes similar to those transformed with *PmLEC1*. T1 desiccation-tolerant seeds were obtained at a transformation frequency of 1.6%. Twenty-five percent of the transgenic plants exhibited normal morphology (Figure 2A) and growth characteristics identical to those of untransformed wild type plants (Figure 2B), whereas other seedlings displayed various abnormalities upon germination, including plant death (Figure 2C–G). As with the *PmLEC1* transgene, some seedlings developed a callus with embryo-like structures (Figure 2G). Calli that multiplied on selection medium for over a year and regenerated shoots were also observed (not shown).

### 2.2. Ectopic Expression of PmLEC1 in the lec1-1^PmLEC1^ T2 Generation Induces Embryonic Programs and Spontaneous Formation of Embryo-like Structures

To confirm whether ectopic expression of *PmLEC1* in *Arabidopsis* leads to the production of somatic embryos in the second generation (T2), 24 individual T1 plants with normal morphology were transferred to the greenhouse to mature, self-pollinate and produce T2 seeds.

Of 24 T1 plants, 14 were found to be sterile and did not produce progeny. Desiccated seeds from only 6 of 10 fertile lines germinated on selective medium. The *lec1-1^PmLEC1^* T2 seeds germinated at a rate of ~25% (13/50); however, only ~10% of the seedlings developed beyond the cotyledon stage. Many seeds reached a standstill after testa rupture (too numerous to count). Several developing seedlings stopped shoot elongation and growth shortly after germination.

The seedlings from five of the six germinated lines developed masses of embryo-like structures (Figure 3) that could be classified as recurrent embryogenesis. These embryo-like structures originated from the cotyledons or the shoot apical meristem region. Numerous structures resembling somatic embryos at the late globular stage were visible, and their numbers increased continuously. Recurrent SE is characterized by the spontaneous appearance of somatic embryos, including complete or partly fused embryo axes and fused cotyledons, and differs from the mode of embryogenesis in which pro-embryogenic masses (PEM) are propagated [9]. Green, organized structures resembling the green embryogenic clusters described by Mordhorst et al. [9] were also observed.

Only one of the six transgenic lines (*lec1-1^PmLEC1^* T2) produced plants capable of wild type vegetative growth (Figure 4A). The wide range of phenotypes exhibited by this line made it a good case for analyzing the involvement of *PmLEC1* in the phenotype and its effects at the level of transcription. Seedlings 6, 7 and 8 (Figure 4A) were morphologically similar to the seedlings with recurrent embryo-like structures in Figure 3. PCR analysis confirmed the presence of the *PmLEC1* transgene and the absence of *AtLEC1* in the genome of this line (Figure 4B). RNA was isolated from leaves, stems, callus-like tissue and roots, but not flowers or siliques, since they may contain developing seeds, in which the expression of embryo-specific genes could confound the results. The isolated RNA was treated with DNase, and RT-PCR was carried out to assess the expression of the *PmLEC1* transgene, as well as the embryo-specific genes, *OLEOSIN* and *CRUCIFERIN* (Figure 4C). Because wild type and *lec1-1* null mutant plants do not express *OLEOSIN* and *CRUCIFERIN* during vegetative growth, the presence of these transcripts confirmed the activation of embryonic programs in the T2 *lec1-1^PmLEC1^* plants (Figure 4C).

All transgenic plants expressed varying levels of *PmLEC1*, whereas wild type and untransformed *lec1-1* null mutant plants did not express any *LEC1* transcripts (Figure 4C). Transgenic seedlings did not express *AtLEC1* (Figure 4C). Plants 1 and 2 did not possess any callus tissue or embryo-like structures and closely resembled normal, vegetative development of *Arabidopsis* plants (Figure 4A). Transgenic plants with the greatest embryo-like character (plants 4, 5, 6, 7 and 8) expressed *OLEOSIN* and *CRUCIFERIN*, while those showing normal plant development (plants 1 and 2) did not express the seed-specific genes (Figure 4A,C). Plant 3 was unusual in that it started out as a callus-like mass, followed by normal development (Figure 4A). At the time of analysis, it showed abundant *OLEOSIN* but negligible *CRUCIFERIN* levels (Figure 4A,C). *PmLEC1* expression was not proportional to the abnormal phenotype: in some cases, comparable levels of transgene expression had opposite effects, for example plants 2 and 7 (Figure 4C). The levels of *PmLEC1* expression (Figure 4C) were confirmed by repeated RT-PCR analyses.

**Figure 5:**
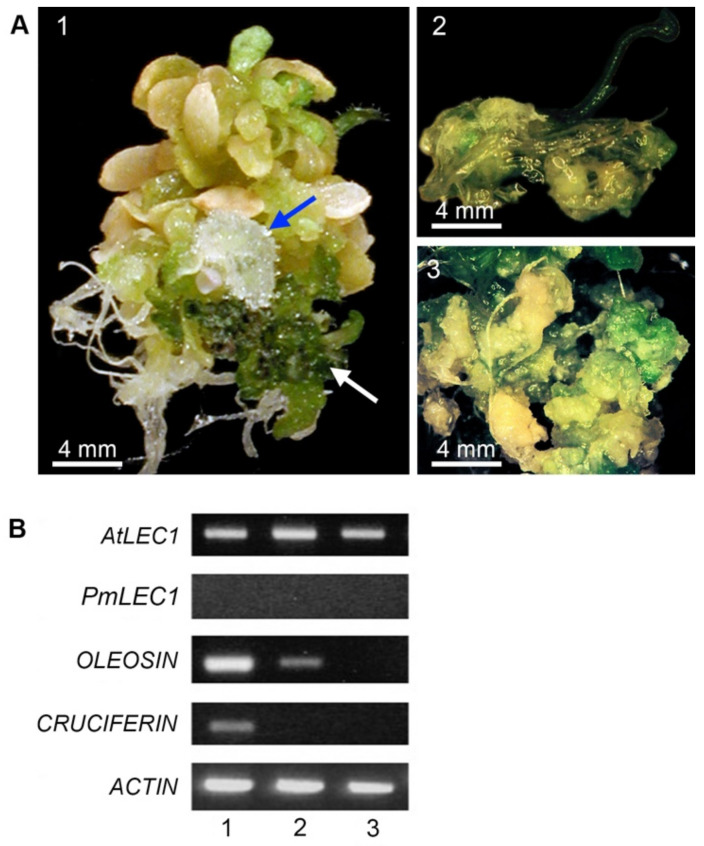
Morphology and gene expression profiles of *Arabidopsis lec1-1^AtLEC1^* T2 seedlings show similarities to *lec1-1^PmLEC1^* T2. (**A**) Morphological variants 1, 2 and 3 of the *AtLEC1*-containing transgenic line. Blue arrow indicates somatic embryo-like masses. White arrow indicates dark green cotyledon-like structures. (**B**) RT-PCR analysis of gene expression. Each reaction comprised 0.1 μg DNase I-treated total RNA isolated from the indicated plant material. The analyzed genes are provided to the left of the corresponding panels. Lanes 1, 2 and 3 represent transcripts from the respective morphological variants 1, 2 and 3, shown in panel A.

In the parallel experiment, ectopic expression of *AtLEC1* in *lec1-1^AtLEC1^* T2 plants arrested vegetative development but did not necessarily activate embryonic programs. Of the 24 *lec1-1^AtLEC1^* T1 plants, 15 were sterile, while 9 flowered and produced seeds. Seeds from only three lines germinated, and less than 1% developed into plants with normal morphology. Most of the *lec1-1^AtLEC1^* T2 seedlings remained in the embryo-like stage, i.e., normal leaves and flowers never developed (Figure 5A). The presence of the transgene was confirmed by PCR. Expression of the transgene and seed-specific genes was analyzed by RT-PCR (Figure 5B). Plant 1 was the most unique: a stack of fleshy, leaf-like structures with a section of dark-green cotyledon-like structures. A mass of recurrent, embryo-like structures emerged from one of the fleshy leaves (Figure 5A, plant 1).

Ectopic expression of *AtLEC1* resulted in abnormal, embryo-like (Figure 5A, plant 1) and callus-like seedlings (Figure 5A, plant 2 and plant 3), activation of embryonic programs (Figure 5B) and the appearance of embryo-like structures (Figure 5A, plant 1). However, embryo-specific gene expression (Figure 5B) was not uniform in all plants with the abnormal phenotype.

### 2.3. Overexpression of PmLEC1 in Wild Type Plants Reduces Apical Dominance but Does Not Activate Embryonic Programs

To gain further insight into the function of *PmLEC1* and assess its ability to induce embryonic programs and somatic embryogenesis in mature, non-mutant plants, we analyzed its overexpression in wild type *Arabidopsis*. First generation transgenic seedlings obtained following transformations of wild type plants with either *PmLEC1* or *AtLEC1* did not display any morphological differences when compared with wild type. Selection on kanamycin-containing MS medium resulted in 2% (4/200) transformation frequency. The T1 plants were transferred to soil and grown in the greenhouse. All transgenic plants produced seeds.

**Figure 6:**
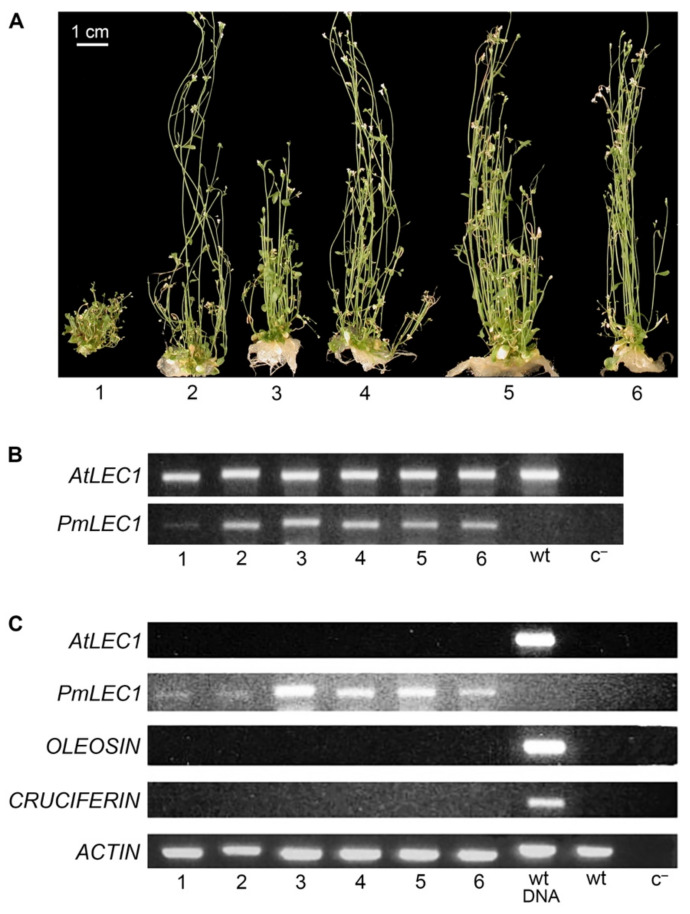
Morphology, genotype analysis and gene expression profiles of individual T2 variants derived from one *wt^PmLEC1^* T1 progenitor line. (**A**) Bushy phenotypes of T2 seedlings derived from one *wt^PmLEC1^* T1 progenitor line. (**B**) PCR analysis of T2 variants using *AtLEC1*- and *PmLEC1*-specific primers (top and bottom panel, respectively) to confirm genotype and the presence of transgenes. Lanes 1 to 6 represent DNA from plants shown in panel A; wt, DNA from a wild type *Arabidopsis* plant (positive control); c^−^, PCR without DNA template (negative control); (**C**) RT-PCR analysis of gene expression. Each reaction comprised 0.1 μg DNase I-treated total RNA isolated from the indicated plant material. The analyzed genes are provided to the left of the corresponding panels. Lanes 1 to 6 represent transcripts from the plants shown in panel A; wt DNA, genomic DNA from a wild type *Arabidopsis* plant (DNA control); wt, RNA from a wild type *Arabidopsis* plant (control, non-transgenic); c^−^, reverse transcription without RNA, followed by PCR (negative control).

The *wt^PmLEC1^* T2 seeds germinated on selective medium, and about 25% possessed kanamycin resistance. Initially, the seedlings started out as a green callus-like mass, but normal vegetative growth resumed within 2–3 weeks. These plants produced leaves and flowers and generally resembled wild type plants, apart from numerous bolts, which caused them to be bushier (Figure 6A). No embryo-like morphology was observed. The *wt^AtLEC1^* T2 generation produced only normal seedlings (not shown).

Only vegetative tissues, stems and leaves were utilized for molecular analyses. All plants contained endogenous *AtLEC1* and the *PmLEC1* transgene (Figure 6B). While all plants expressed the *PmLEC1* transgene within mature tissues, none expressed *AtLEC1* or the embryo-specific genes *OLEOSIN* and *CRUCIFERIN* (Figure 6C); therefore, no embryonic programs were activated in the vegetative tissues. Thus, overexpression of *PmLEC1* in wild type plants did not induce embryonic pathways in vegetative cells and did not lead to spontaneous formation of embryo-like structures.

### 2.4. PmLEC1 Protein Accumulation in Transgenic Plants Correlates with Embryo-like Character and Suppression of Vegetative Development

**Figure 7:**
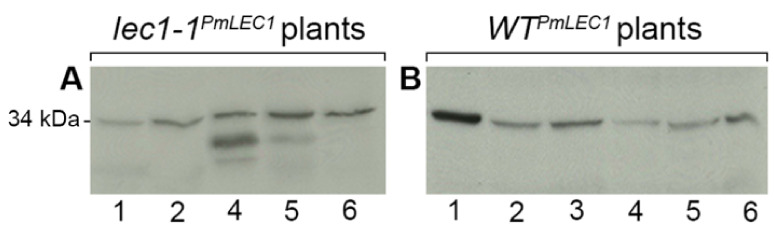
Western blot analysis of heterologous PmLEC1 peptide accumulation in transgenic *Arabidopsis* T2 plants. Total protein lysates were isolated from (**A**) second generation transgenic *lec1-1* null mutant plants transformed with *PmLEC1* (lane numbers correspond to the plants in Figure 4A) and (**B**) second generation wild type plants transformed with *PmLEC1* (lane numbers correspond to the plants in Figure 6A). The protein samples (20 μg) were separated by SDS-PAGE, transferred to a PVDF membrane and incubated with polyclonal anti-PmLEC1 serum diluted 1:1000 for 1 h.

Antibodies were raised against a synthetic peptide corresponding to the *N*-terminus of PmLEC1 and validated by ELISA and western blot analysis against free peptide and cellular proteins extracted from developing Douglas-fir seeds, as well as *Arabidopsis* wild type, *lec1-1* null mutant and transgenic plants [6]. Total proteins were extracted from the *lec1-1^PmLEC1^* T2 plants shown in Figure 4A and the *wt^PmLEC1^* T2 plants shown in Figure 6A. Western blot analysis was performed to determine the relationship between PmLEC1 accumulation and the observed phenotypes (Figure 7). Stronger immunoreactive bands were observed at 34 kDa in transformed *lec1-1* plants showing the greatest embryo-like character and in transformed wild type plants showing reduced apical dominance (Figure 7A lanes 4, 5, 6; Figure 7B, lanes 1 and 3). Weaker immunoreactive bands were observed in all transformants containing and expressing the *PmLEC1* gene.

Although all plants expressed *PmLEC1* transcripts to varying degrees (Figure 4C and Figure 6C), protein accumulation did not correspond to RNA levels. For example, wild type transformants 1 and 2 (Figure 6A) both showed very low *PmLEC1* gene expression (Figure 6C); however, phenotypically, plant 1 exhibited a severe loss of apical dominance and high PmLEC1 protein accumulation (Figure 7B); thus, PmLEC1 protein accumulation appears to be a better indicator of phenotypic effects.

## 3. Discussion

### 3.1. Douglas-Fir PmLEC1 Is Functionally Homologous to Arabidopsis LEC1 and Induces Embryonic Programs in the Arabidopsis lec1-1 Null Mutant

In complementation analysis of the *lec1-1* null mutant, *PmLEC1* displayed similar function to *AtLEC1*. When expressed ectopically in the *lec1-1* null mutant, either gene rescued the null mutant and induced embryonic programs and somatic embryo-like structures (Figure 1 and Figure 2). In the T2 generation, ectopic expression of each gene resulted in seedlings with abnormal morphology, representing an intermediate state between embryonic and vegetative development (Figure 4 and Figure 5). These results are in agreement with the work of Lotan et al. (1998), who observed embryo-like structures on vegetative tissues of the *lec1-1* null mutant transformed with *AtLEC1* [2]. A difference we found between *lec1-1^AtLEC1^* and *lec1-1^PmLEC1^* was that in the T2 generation, *AtLEC1* transformants never escaped the embryo-like character and never produced progeny. This could be because the *35S-35S-AMV* promoter is very strong and AtLEC1 protein has a more specific function in *Arabidopsis*, while PmLEC1, being non-species-specific, may be less effective in developmental arrest and more permissive to vegetative development. *AtLEC1* induces embryo maturation programs and suppresses vegetative development [2]; thus, strong, constitutive *AtLEC1* expression is expected to prevent normal vegetative development. At the amino acid level, PmLEC1 shows only 56% identity to AtLEC1; thus, its interaction with other NF-Y transcription factor subunits in *Arabidopsis* may not be as strong or specific.

Another notable difference between *lec1-1^AtLEC1^* and *lec1-1^PmLEC1^* plants was the timing of the embryo-like structure generation. In *PmLEC1* transformants, somatic embryo-like structure formation started from the cotyledons shortly after radicle formation. In *AtLEC1* transformants, on the other hand, the appearance of embryo-like structures took place after multiple cotyledon-like organs had formed. Again, this difference may be attributed to the primary amino acid sequence and the possibility that AtLEC1 and PmLEC1 have slightly different interactions with the other transcription factor subunits.

The embryo-like structures we observed were similar to those that are produced when SE is induced via 2,4-D treatment of *Arabidopsis* heart stage zygotic embryos [10]. The recurrent embryo-like pattern of T2 *lec1-1^PmLEC1^* (Figure 3 and Figure 4) strongly resembled the somatic embryo induction pattern of *primordia timing* (*pt*) and *clavata* (*clv*) mutants, which exhibit an enhanced embryogenic phenotype and are characterized by an enlarged shoot apical meristem, embryogenic cluster formation from seedlings treated with 2,4-D, maintenance of embryogenic capacity for two years in sub-culture, polycotyly and, at the plant level, an increased number of rosette leaves, fasciation and an increased number of side shoots [9].

Plants with reduced apical dominance often display a bushy phenotype. The *Arabidopsis pt* mutant has an enhanced embryogenic phenotype due to reduced apical dominance and produces an excessive number of side shoots [9]. Since one function of *AtLEC1* is to prevent precocious germination during embryogenesis [2], overexpression of *LEC1* genes may also suppress apical dominance.

In our study, PmLEC1 protein accumulation in transgenic plants did not correlate with *PmLEC1* gene expression. This is not surprising since a variety of mechanisms (e.g., protein degradation, stabilization, sequestration, translation rate modulation) can contribute to discord between transcript levels and protein levels [11]. However, the protein levels strongly correlated with plant phenotype in this work, indicating that the use of anti-PmLEC1 antibodies may provide a more accurate estimate of PmLEC1 cellular activity.

### 3.2. Transgene Silencing May Account for the Episodes of Normal Development Observed in lec1-1^PmLEC1^ T1 and T2 Transgenic Plants

The diverse phenotypes of *lec1-1^PmLEC1^* T1 and T2 plants observed in this study may be the result of post-transcriptional gene silencing and subsequent reactivation, either due to progeny receiving non-methylated strands when the coding sequence was hemi-methylated [12], or when methylation is reset during meiosis [13]. Additionally, the range of phenotypes could be a result of multiple transgene insertions or position effects [14]; a high number of transgene copies per genome above a critical threshold activates a sequence-specific degradation system and degrades the mRNA, resulting in posttranscriptional transgene silencing [15,16]. Transgene activity in T1 seeds rescued the *lec1-1* null mutant by conferring desiccation tolerance and producing viable, mature seeds. The T1 seeds gave rise to T1 plants, and post-transcriptional gene silencing may have occurred in some T1 plants. These plants grew vegetatively, and reversal of gene silencing at meiosis permitted some developing T2 seeds to reach maturity. Viable T2 seeds gave rise to T2 plants, which showed a variety of phenotypes, ranging between vegetative development and developmental arrest (Figure 4). Transgene silencing and reactivation may explain the gene expression profiles of *lec1-1^PmLEC1^* T2 plants 1, 2 and 3 (Figure 4); silencing led to a loss of seed-specific gene expression, as in 1 and 2, while plant 3 was in the process of escaping developmental arrest because a few shoots developed but an embryo-like callus was still a significant part of the seedling. High-level *OLEOSIN* expression combined with low-level *CRUCIFERIN* expression and normal vegetative character may represent a transitional phase wherein embryonic programs are being shut down and vegetative growth begins (Figure 4C, lane 3). It is possible that plants 4 and 6 (Figure 4) were in the process of transgene silencing, with *PmLEC1* expression being down-regulated and a single shoot arising from the callus-like tissue in plant 4, and potentially the beginning of vegetative growth for plant 6. Plants 5, 7 and 8 were developmentally arrested and actively expressing the transgene and seed-specific genes, exhibiting an embryo-like (5) or callus-like (7 and 8) phenotype.

The decreasing number of viable transgenic plants obtained in the T2 generation were likely the result of pre-transcriptional gene silencing, mediated via the RNAi pathway by inducing heterochromatin formation [17,18]. Silencing of the transgene before completion of seed maturation would re-establish the *lec1-1* phenotype and produce non-viable seeds, which would not germinate on any medium. Alternatively, temporary transgene silencing would allow normal development to take place in the presence of the transgene, while reactivation at meiosis would allow transgenic *lec1-1* seeds to regain viability.

### 3.3. PmLEC1 May Function Prior to Embryogenesis

More extreme phenotypes were observed in the T2 generations of both *lec1-1* and *wt* transformants, suggesting that *PmLEC1* has activity prior to fertilization or embryogenesis. In the floral dip method, the transgene is delivered at the time of fertilization, and any activity or effects that the transgene may have had prior to that time will not be evident in the T1 generation. The callus mass formation and bushiness of the T2 generation of *wt^PmLEC1^* plants but not its T1 generation, and the rare occurrence of embryo-like structures in *lec1-1^PmLEC1^* T1 (Figure 1) compared to the recurrent formation of embryo-like cell masses and developmental arrest in *lec1-1^PmLEC1^* T2 (Figure 3 and Figure 4), suggest that LEC1 has activity prior to flowering and fertilization. In Douglas-fir, *PmLEC1* RNA and protein expression are observed in the unfertilized ovule [6], further supporting a role for *PmLEC1* prior to fertilization. Additionally, it is not clear that *AtLEC1* is not expressed in pre-embryonic tissues of *Arabidopsis* due to the low sensitivity of northern blotting in the early work on *AtLEC1*. Finally, bioinformatic analysis suggests that *PmLEC1* may be regulated by transcription factors that function in flowering, pollen tube and ovule development [6]. The difference in outcomes when *LEC1* is expressed prior to embryogenesis implies that *LEC1* may have long-term effects on the overall outcome of development, and that expression of *LEC1* prior to embryogenesis may permit induction of SE.

### 3.4. LEC1 by Itself Cannot Induce De Novo Embryogenesis

Comparison of *lec1-1^PmLEC1^* T2 with *wt^PmLEC1^* T2 suggests that *LEC1* by itself is not solely responsible for the embryo-like phenotype observed with the *lec1-1* transformants. In the *lec1-1* null mutant, ectopic expression of *PmLEC1* in mature and germinating seeds resulted in callus masses and spontaneous formation of embryo-like structures (Figure 3). However, ectopic expression of *PmLEC1* in wild type vegetative tissues or wild type germinating seeds did not have the same embryogenic effect despite a strong constitutive promoter. Some of the *lec1-1^PmLEC1^* T2 plants exhibited normal vegetative development (plants 1 and 2 in Figure 4A), as did all wild type T2 plants (Figure 6A). While these vegetatively growing plants expressed the transgene, the absence of *CRUCIFERIN* and *OLEOSIN* transcripts in *lec1-1^PmLEC1^* plants 1 and 2 and in all *wt^PmLEC1^* plants (Figure 4C and Figure 6C) indicates that embryo-specific programs were not induced. This was also corroborated by the normal phenotype of the plants. Moreover, all the transformants of wild type genetic background started from a seed-derived callus, which eventually led to shoot and root formation, instead of following the normal developmental pathway of seed germination followed by shoot growth. This suggests that PmLEC1 modified development within the seed environment, when other seed-specific transcription factors were active, but in the end, a stronger program ensuring normal plant vegetative growth negated its effects. A plausible explanation is that spontaneous embryogenesis occurs under specific developmental cues and when LEC1 can interact with other embryogenic factors within the seed. LEC1 is a transcription factor subunit and requires the presence of HAP2 and HAP5, or other partners to activate embryogenic programs.

The lack of correspondence between *PmLEC1* levels and *OLEOSIN/CRUCIFERIN* levels supports the need for specific subunit interactions. Some embryo-like seedlings expressed lower levels of *PmLEC1*, but *OLEOSIN* and *CRUCIFERIN* expression was not proportional. In plant 4 of the *lec1-1^PmLEC1^* T2 (Figure 4C), *PmLEC1* expression was low, while *CRUCIFERN* and *OLEOSIN* levels were high. Conversely, in plant 5 (Figure 4C), *PmLEC1* expression was high but *CRUCIFERIN* expression was very low. Overall, PmLEC1 protein accumulation was higher in *lec1-1^PmLEC1^* T2 plants expressing both *OLEOSIN* and *CRUCIFERIN* (Figure 4C and Figure 7A). The *wt^PmLEC1^* T2 plants also showed various degrees of *PmLEC1* expression, and while this was comparable to the *lec1-1^PmLEC1^* T2 seedlings in some cases (plant 5 in Figure 4C, plant 3 in Figure 6C), none of the wild type plants expressed *CRUCIFERIN* or *OLEOSIN* (Figure 6C). Moreover, similar amounts of *PmLEC1* transcript or protein were sometimes observed in both abnormal embryo-like seedlings and plants with normal phenotype. Plant 1 of the *wt^PmLEC1^* T2 generation accumulated a markedly high level of PmLEC1 protein and exhibited severe phenotypical abnormalities, although *CRUCIFERIN* and *OLEOSIN* were not expressed in this plant (Figure 6 and Figure 7). Hence, neither a high abundance of *LEC1* transcripts nor excessive LEC1 protein levels were sufficient for the induction of embryonic programs in vegetative tissues beyond the seedling stage.

The inconsistency of embryo-specific gene expression was also observed in *lec1-1^AtLEC1^* T2 plants (Figure 5B) and may indicate that some of these callus-like seedlings were not embryogenic. Thus, expression of *AtLEC1* arrested normal vegetative development but did not always induce somatic embryogenesis.

Our findings suggest that ectopic expression of *LEC1* by itself cannot induce de novo embryogenesis, but the interaction of LEC1 with factors present in the seed may lead to an embryo-like character and embryogenesis in seeds in which maturation is disrupted, such as *lec1-1* seeds. With respect to conifer embryogenesis, disruption of seed maturation by incubation on plant growth regulator medium may be the mechanism by which PmLEC1 induces SE. Up-regulation of PmLEC1 protein in recalcitrant genotypes may improve SE initiation rates because LEC1 specifies embryonic organ identity. Thus, an induction medium containing both plant growth regulators (PGRs) and compounds that specifically induce *LEC1* expression may be a novel strategy for inducing SE in conifers.

### 3.5. The AtLEC1 Promoter Region May Exert Control over Embryogenesis

Ectopic expression of *PmLEC1* in wild type plants did not have the same dramatic effect as in *lec1-1* null mutant plants; the *AtLEC1* promoter region may be responsible for preventing an embryo-like character in wild type transformants. The *lec1-1* null mutant lacks both the *AtLEC1* gene and the *AtLEC1* promoter. The *lec1-1^PmLEC1^* T2 generation was characterized by recurrent somatic embryo-like structures, developmental arrest and induction of embryonic programs, while the *wt^PmLEC1^* T2 generation was characterized by callus-like masses forming from the cotyledons shortly after germination, followed by development of plants that were bushier than the wild type. Embryonic programs were not induced in transgenic plants of the wild type background. The presence of the callus-like masses suggests that PmLEC1 interacted with other factors that were present within the germinating seed and stimulated proliferative programs. The subsequent appearance of shoots, the growth of plants and the absence of embryo-specific gene transcripts suggest that something occurred to inhibit the proliferative state and promote vegetative growth. The vegetatively growing plants expressed *PmLEC1* transcripts but not the seed specific RNAs for *OLEOSIN* and *CRUCIFERIN*. The use of a strong constitutive promoter and the appearance of cell masses resembling somatic embryos in the *lec1-1^PmLEC1^* T2 plants led to the expectation that *wt^PmLEC1^* T2 plants would also show embryo-like character and somatic embryo-like structure production. Since this did not happen, the *AtLEC1* promoter that was present in *wt^PmLEC1^* and *wt^AtLEC1^* plants, but absent in *lec1-1^PmLEC1^* and *lec1-1^AtLEC1^*, may have been responsible for terminating the embryogenic program, effectively suppressing the proliferative state and preventing somatic embryogenesis.

Evidence for the above type of promoter activity comes from the *turnip* (*tnp*) mutant of *Arabidopsis*, in which the promoter region of *AtLEC1* is deleted, resulting in a gain-of-function mutation for *AtLEC1* [16]. Not only is *AtLEC1* upregulated in this mutant, but *P1P5K*, which is adjacent and downstream of *AtLEC1*, is also upregulated. It was suggested that the *AtLEC1* promoter region contains a coordinating mechanism for the repression of both genes [19]. Moreover, DNA sequences located 1200 bp upstream of the *Arabidopsis ISOCITRATE LYASE* gene have a major role in its activation, and the 3500 bp sequence upstream of the gene is sufficient to specify expression at different developmental stages [20]. Thus, if the *AtLEC1* promoter regulates and silences expression of a few essential factors required for embryogenesis, perhaps the entire process may be shut down. A coordinating mechanism within the promoter of *LEC1* potentially explains our results with wild type transformants, namely the observed transition from callus-like growth to vegetative development (Figure 6A). This finding is significant for conifer species, because SE induction in gymnosperms is difficult and many genotypes are recalcitrant to SE induction. Perhaps future examination of the upstream regulatory sequences of *PmLEC1* will provide insights that will allow meaningful progress for conifer SE.

In conclusion, our study has demonstrated that Douglas-fir *PmLEC1* rescued the *Arabidopsis lec1-1* null mutant and induced embryonic programs and formation of embryo-like structures in both T1 and T2 generations. Transcripts for seed-specific *OLEOSIN* and *CRUCIFERIN* were observed in the embryo-like seedlings. In transformations of wild type *Arabidopsis*, *PmLEC1* had no visible effect on the T1 generation but produced a bushy phenotype in the T2 generation. The *wt^PmLEC1^* T2 plants expressed *PmLEC1* transcripts but not the embryo-specific RNAs for *OLEOSIN* and *CRUCIFERIN*. Because the T2 generations showed a more severe phenotype and the *PmLEC1* transgene was available throughout the lifecycle in T2 but not in T1, we postulate that the activity of LEC1 prior to embryogenesis influences the course of embryogenesis. Although *PmLEC1* gene expression and protein accumulation were observed in *wt^PmLEC1^* T2, the absence of embryo-like morphology and embryo-specific transcripts suggests that the *AtLEC1* promoter controls other genes involved in embryogenesis. Additional work with overexpression in wild type *Arabidopsis* and assessment of embryonic gene expression at shorter time intervals will delineate the exact stages at which *LEC1* overexpression results in callus formation and when vegetative development resumes. For conifers, the *LEC1* gene has critical roles in embryogenesis both at the functional and regulatory levels, and the PmLEC1 protein is a potential biomarker of SE.

## 4. Materials and Methods

### 4.1. Plant Material

Wild-type and heterozygous Lec1/lec1 seeds of *Arabidopsis thaliana* (L.) Heynh ecotype Wassilewskija were obtained from the *Arabidopsis* Biological Resource Center (ABRC) at the Ohio State University (ABRC Stock Numbers CS2360 and CS8101, respectively). Heterozygous seeds (*LEC1/lec1*) were germinated and grown in pots under standard greenhouse conditions. The plants were self-pollinated to generate homozygous progeny. Immature seeds were removed from green siliques, surface sterilized and plated in vitro on semi-solid half-strength MS medium [21]. Only homozygous *lec1-1* seeds germinate under these conditions. Immature wild type seeds do not germinate precociously. The germinated *lec1-1* seeds were grown into plantlets and the genotype was confirmed by PCR using *AtLEC1* gene specific primers.

### 4.2. Construction of Expression Cassettes

Two expression cassettes were constructed: one contained the *PmLEC1* gene and another contained the *AtLEC1* gene. PCR amplification of the *PmLEC1* and *AtLEC1* coding sequences was carried out with primers containing additional sequence for *Xba*I and *Bam*HI recognition sites at the 5′ and 3′ ends of each gene, respectively. The PCR product was digested with the corresponding restriction enzymes, and the transgene was directionally inserted into the *Xba*I and *Bam*HI sites of the pBI121 vector [22] between the *35S-35S-AMV* promoter and the *NOS* terminator. The vector contained the *neomycin phosphotransferase II* (*NPT II*) gene for kanamycin resistance. The resulting vectors were transferred into *E. coli*, and the integrity of the inserts was confirmed by DNA sequencing. The vectors were transferred into *Agrobacterium tumefaciens* strain MP90 according to Datla [9]. The transformed cells were selected in antibiotic-containing medium and the presence of the insert was confirmed by restriction analyses.

### 4.3. Agrobacterium-Mediated Plant Transformation

The *Arabidopsis lec1-1* null mutant and wild type plants were transformed using the floral dip method described by Clough and Bent [23]. Flowering plants (T0 generation) were dipped into *Agrobacterium* solution and produced the first transgenic generation seeds (T1). Transgenic plants were selected from T1 seeds germinated on semi-solid MS medium [18] containing 100 mg/L kanamycin sulfate, and transgene integration was confirmed by PCR using gene-specific primers. The floral dip method of transformation produces transformants (T1) that are hemizygous at any T-DNA insertion site. T1 plants were self-pollinated to produce T2 seeds. T2 seeds were germinated on kanamycin-containing MS medium to select T2 plants, which were homozygous or hemizygous at the transgene insertion site.

### 4.4. DNA Isolation and PCR Analysis

Plants were ground in liquid nitrogen, and DNA was isolated using the Sigma GenElute Plant Genomic DNA kit (Sigma, St. Louis, MO, USA) according to the manufacturer’s instructions. PCR reactions were performed in 50 μL reactions containing 0.5 μg DNA and Taq PCR Master Mix (Qiagen, Toronto, ON, Canada) according to the manufacturer’s instructions. The products were separated by electrophoresis in a 1% agarose gel and visualized by ethidium bromide staining.

For *AtLEC1* amplification, the primers utilized were forward 19-mer 5′-ATGACCAGCTCAGTCATAG-3′ and reverse 21-mer 5′-TCACTTATACTGACCATAATG-3′, which generate a 624 bp product. The thermocycle program consisted of a 5 min denaturation at 94 °C, 40 cycles of denaturation at 94 °C for 30 s, annealing at 51 °C for 30 s and extension at 72 °C for 1 min, followed by 10 min of extension at 72 °C.

*PmLEC1* was amplified using the primers 5′-ATGATGTCCGAAGTTGGAAGC-CCT-3′ and 5′-CTTATACTGAGCATAGGGATCATA-3′, which generate a 540 bp product. The thermocycle program consisted of 5 min denaturation at 94 °C, 40 cycles of denaturation at 94 °C for 30 s, annealing at 61 °C for 30 s and extension at 72 °C for 1 min, followed by 10 min of extension at 72 °C.

*CRUCIFERIN* was amplified using primers 5′-ATGGTGCTTCCTAAATACAAG-3′ and 5′-TTAAGCCTCGACAATCTCCT-3′, which generate a 382 bp product. The thermocycle program consisted of 5 min denaturation at 94 °C, 40 cycles of denaturation at 94 °C for 30 s, annealing at 53 °C for 30 s and extension at 72 °C for 1 min, followed by 10 min of extension at 72 °C.

*OLEOSIN* was amplified using primers 5′-ATGGCCGATACAGCTAGAGG-3′ and 5′-AGAGAAAACGGTTATAGCGGC-3′, which generate a 321 bp product. The thermocycle program consisted of 5 min denaturation at 94 °C, 40 cycles of denaturation at 94 °C for 30 s, annealing at 58 °C for 30 s and extension at 72 °C for 1 min, followed by 10 min of extension at 72 °C.

Wild type *Arabidopsis* DNA was a positive control for *AtLEC1*, *CRUCIFERIN* and *OLEOSIN* amplifications. No-template negative controls were performed for all reactions.

### 4.5. RNA Isolation and RT-PCR Analysis

Tissue used for RNA isolation excluded flowers, seeds and siliques in order to eliminate the possibility that seed of any stage that could show expression of seed storage proteins, CRUCIFERIN and OLEOSIN. Total RNA was isolated by the TRIzol (Invitrogen, Waltham, MA, USA) method, modified for plants according to the manufacturer’s instructions. RNA isolated from all samples was treated with amplification grade DNase I (Invitrogen, Waltham, MA, USA) according to the manufacturer’s instructions.

For first strand cDNA synthesis, 1 μg total RNA was incubated with 1 μL oligo (dT)_12_VN (V = A or C or G; N = A or C or G or T) and SuperScript II RNase H^−^ reverse transcriptase in 20 μL reactions (Invitrogen, Waltham, MA, USA) according to the manufacturer’s instructions. The Invitrogen Ribonuclease Inhibitor (1 μL at 10 U/μL) was utilized to prevent RNA degradation in the reactions. PCR was performed in 25 μL reactions containing 2 μL cDNA and Taq PCR Master Mix (Qiagen, Toronto, ON, Canada), as described above.

### 4.6. Western Blot Analysis

A peptide corresponding to the first 18 amino acids of the putative PmLEC1 protein, with an additional cysteine residue at the C-terminus (MMSEVGSPTSQDSRNSEDC) and coupled to the KLH carrier protein, was synthesized by GenScript Biotech Corporation (Piscataway, NJ, USA). Antibody production was performed at ImmunoPrecise Antibodies, Ltd. (Victoria, BC, Canada). Four Balb/C mice were each immunized with 25 μg of the KLH-coupled peptide, mixed with Freund’s complete adjuvant. Six additional immune boosts of 25 μg peptide-KLH in Freund’s incomplete adjuvant followed at 3-week intervals. Dilutions of the polyclonal mouse antiserum were tested by ELISA against the free peptide and protein extracts from Douglas-fir developing seed. The polyclonal antiserum from two mice showed a significant response against the peptide when used at a dilution of 1:1000. Blood was drawn from these 2 mice on four dates over a 2-month period. The antisera were obtained by centrifugation and combined for a total of 6 mL of polyclonal antiserum used for western blot analysis.

Total proteins were extracted from frozen and ground stem and leaf tissues and suspended in extraction buffer (65 mM Tris [pH 6.8], 1% SDS, 5% glycerol and 2.5% β-mercaptoethanol) at 1 mg per 3 μL. Protein concentrations were determined by the Bradford assay [24].

Total proteins (20 μg) were resolved by SDS-PAGE and transferred to PVDF membranes (GE Healthcare, Chicago, IL, USA). Equal protein loading was confirmed by staining duplicate gels with Coomassie brilliant blue. Western blot analysis was performed with mouse anti-PmLEC1 primary antibody (ImmunoPrecise Antibodies), diluted 1:1000, and ImmunoPure Goat Anti-Mouse IgG, Peroxidase Conjugated (Pierce Biotechnology, Waltham, MA, USA) secondary antibody, diluted 1:100,000. Proteins were detected by chemiluminescence with ECL Plus Western Blotting Detection Reagents (GE Healthcare, Chicago, IL, USA).

## Data Availability

The data presented in this study are available on request from the corresponding author.
